# Childhood Immunization Coverage Before, During and After the COVID-19 Pandemic in Italy

**DOI:** 10.3390/vaccines13070683

**Published:** 2025-06-25

**Authors:** Flavia Pennisi, Andrea Silenzi, Alessia Mammone, Andrea Siddu, Anna Odone, Michela Sabbatucci, Riccardo Orioli, Anna Carole D’Amelio, Francesco Maraglino, Giovanni Rezza, Carlo Signorelli

**Affiliations:** 1 National PhD Programme in One Health Approaches to Infectious Diseases and Life Science Research, Department of Public Health, Experimental and Forensic Medicine, University of Pavia, 27100 Pavia, Italy; pennisi.flavia@hsr.it; 2 School of Medicine, Università Vita-Salute San Raffaele, 20132 Milan, Italy; carole.damelio@gmail.com (A.C.D.); rezza.giovanni@hsr.it (G.R.); signorelli.carlo@hsr.it (C.S.); 3 Center for Research and Studies on Leadership in Medicine, Università Cattolica del Sacro Cuore, 00168 Rome, Italy; 4 General Directorate of Health Prevention, Ministry of Health, 00153 Rome, Italy; a.mammone@sanita.it (A.M.); r.orioli@sanita.it (R.O.); f.maraglino@sanita.it (F.M.); 5 Health Promotion and Prevention, Regional Directorate for Health and Social-Health Integration, Lazio Region, 00193 Rome, Italy; a.siddu@sanita.it; 6 Department of Public Health, Experimental and Forensic Medicine, University of Pavia, 27100 Pavia, Italy; anna.odone@unipv.it; 7 Italian National Immunization Technical Advisory Group (NITAG), Ministry of Health, 00153 Rome, Italy

**Keywords:** vaccines, mandatory vaccination, vaccination coverage, COVID-19 pandemic, vaccination strategies

## Abstract

**Background/Objectives:** Maintaining high childhood vaccination coverage is essential to prevent outbreaks of vaccine-preventable diseases. In Italy, Law No. 119/2017 introduced mandatory childhood immunizations, leading to significant improvements. However, the COVID-19 pandemic disrupted routine services, potentially jeopardizing these gains. This study aimed to evaluate national and regional trends in vaccine coverage across three phases: post-mandate (2015–2016 vs. 2017–2019), pandemic (2017–2019 vs. 2020–2021), and post-pandemic recovery (2020–2021 vs. 2022–2023). **Methods:** National and regional administrative data on vaccination coverage at 24 months of age were obtained from the Italian Ministry of Health. Temporal trends were analyzed using Joinpoint regression to estimate annual percent changes (APCs), and absolute changes in coverage (Δ) were calculated across defined periods. Pearson correlation coefficients were used to assess associations between baseline coverage and subsequent changes. **Results:** After the 2017 mandate, coverage increased significantly for varicella (APC = +28.6%), MenB (+22.6%), and measles (+3.4%). Regionally, varicella coverage rose by up to +58.4% in Emilia-Romagna and measles by +11.1% in Campania. During the pandemic, coverage declined for polio (−2.4% in the South) and measles (−6.2% in Abruzzo), while MenB increased in regions with lower initial uptake (r = −0.918, *p* < 0.001). Post-pandemic, coverage rebounded, with varicella improving by +20.1% in central regions and measles by +13.9% in Abruzzo. A strong inverse correlation between baseline coverage and improvement was observed for varicella across all periods (r from −0.877 to −0.915). **Conclusions:** Mandatory vaccination policies led to substantial coverage improvements, and despite the disruption caused by the pandemic, recovery trends were observed for most vaccines. The consistent association between low baseline coverage and stronger gains highlights the resilience of the system, but also the ongoing need for regionally tailored strategies to reduce geographic disparities and ensure equitable immunization across Italy.

## 1. Introduction

The emergence of SARS-CoV-2 in late 2019 and its rapid global spread had profound repercussions on healthcare systems, including routine immunization services [1,2]. The reallocation of healthcare resources, mobility restrictions, and heightened public concerns about infection risk contributed to a significant disruption in vaccine delivery and uptake worldwide. In response to these developments, the World Health Organization (WHO) issued interim guidelines warning of the heightened risk of outbreaks caused by vaccine-preventable diseases (VPDs), emphasizing the importance of maintaining essential immunization programs even during public health emergencies [3,4].

In Italy, a country with a historically decentralized health system, the implementation of Law No. 119/2017 marked a critical turning point in immunization policy [5]. The law reinforced the mandate for four vaccines that were already mandatory (polio, diphtheria, tetanus, and hepatitis B) and extended the requirement to six additional vaccines, including pertussis, Haemophilus influenzae type B (Hib), measles, mumps, rubella (MMR), and varicella (chickenpox). Each of Italy’s nineteen regions and two autonomous provinces adopts distinct strategies for the organization and delivery of immunization services [6]. These regional variations are shaped by multiple factors, including the administrative autonomy granted to regional health authorities, the nature and extent of local agreements with healthcare stakeholders, such as general practitioners, community pharmacies, and long-term care facilities, and the availability and distribution of qualified healthcare personnel [7,8,9,10,11].

However, the first wave of the COVID-19 pandemic, which struck Northern Italy with particular severity in early 2020 [10,12,13,14], created substantial obstacles for immunization delivery. The combination of lockdown measures, fear of contagion, and healthcare system saturation negatively affected both vaccine demand and access to services [15,16,17]. A preliminary national analysis comparing coverage rates in 2019 and 2020 revealed a modest decline in coverage at both 24 months and 7 years of age for all mandatory vaccines, except varicella, a mandatory vaccine, which remained stable or slightly increased [18]. A less clear trend in both periods was observed for recommended vaccinations (ACWY, meningococcal B, pneumococcus and anti-rotavirus).

A recent birth cohort analysis from the United States showed that coverage among children born during 2018–2019 increased when compared with coverage among children born during 2016–2017 for a majority of recommended vaccines; overall, coverage was similar or higher among children reaching 24 months during March 2020 or later, compared with the pre-pandemic period [19]. However, declines were observed in certain subgroups and transient declines in measles, mumps, rubella and varicella vaccine coverage for those turning 13 months in March-April 2020, but they were followed by subsequent increases at later ages at 24 months [20].

This study aims to evaluate temporal trends in childhood vaccination coverage in Italy at 24 months of age, across three critical periods: following the 2017 vaccination mandate, during the COVID-19 pandemic, and the initial post-pandemic recovery. Specifically, it investigates national and regional changes in coverage for five key vaccines, polio, measles, varicella, pneumococcal, and meningococcal B (MenB), while exploring regional and geographic disparities and examining whether baseline coverage levels may be associated with differential responsiveness across phases.

## 2. Materials and Methods

### 2.1. Data Source

Anonymized and aggregated vaccination data were obtained from the Italian Ministry of Health, which annually collects information from Local Health Authorities (LHAs) across Italy’s nineteen regions (R) and two autonomous provinces (AP). The dataset includes the number of vaccine doses administered (numerator) and the size of the corresponding resident birth cohort (denominator), allowing for the calculation of coverage rates.

This study focused on vaccination coverage at 24 months of age for five vaccines, selected based on their policy relevance, variability in legal status (mandatory vs. recommended), and epidemiological importance in early childhood: poliomyelitis, measles, varicella (chickenpox), pneumococcal conjugate vaccine, and MenB, covering the birth cohorts from 2013 to 2021, based on data collected from 2015 to 2023. Poliomyelitis and measles were used as proxies for the hexavalent and trivalent vaccines, respectively, given that these antigens are routinely administered in combination formulations (Polio is combined with diphtheria, tetanus, pertussis, Haemophilus influenzae type b, and hepatitis B, and measles with mumps plus rubella, respectively). Varicella, which became mandatory for children born from 2017 onward, is now typically administered in a quadrivalent MMRV vaccine formulation. This allows for the assessment of the mandate’s impact. Pneumococcal and MenB vaccines, which are recommended but not mandatory, were included to assess trends in non-mandatory immunizations and to explore whether they were similarly affected by systemic disruptions such as the COVID-19 pandemic. Other vaccines were excluded due to incomplete or inconsistent regional reporting or because their recommended administration schedule extends beyond 24 months. Additionally, antigens already represented within the analyzed combination vaccines (e.g., rubella and mumps within MMR, or diphtheria and pertussis within the hexavalent formulation) were not considered separately to avoid analytical redundancy.

Data were reported by birth cohort and year of vaccine administration, and referred to children who had completed the full vaccination schedule for each antigen by 24 months of age, regardless of the specific vaccine formulation or immunization schedule used. All analyses were conducted using StataNow/SE 18.5 (StataCorp LLC, College Station, TX, USA).

### 2.2. Joinpoint Regression Analysis of Vaccination Coverage Trends (2014–2021)

Joinpoint regression analysis was conducted to examine temporal trends in national vaccination coverage for each of the five vaccines. The analysis was based on birth cohort data from 2013 to 2021. Using RStudio (version 2024.12.0+467) and the segmented package, we fitted piecewise linear regression models to log-transformed coverage rates.

Separate models were estimated for each vaccine, allowing for up to two joinpoints per model. Joinpoints were identified through iterative model fitting and optimization based on the Bayesian Information Criterion (BIC). Their statistical significance was tested using likelihood ratio tests. For each segment, the Annual Percent Change (APC) and corresponding 95% confidence intervals (CIs) were calculated. Graphical outputs included observed data points, fitted regression lines, and vertical lines indicating joinpoint positions. All analyses were based on complete case data. For Joinpoint regression, only birth cohorts with complete national coverage data were included (2014–2021), and no imputation was applied. When present for specific years or vaccines, missing values led to the exclusion of those time points from the respective models.

### 2.3. Period Definitions

To evaluate temporal changes in vaccination coverage across Italian regions, we analyzed administrative data on childhood immunization rates at 24 months of age for the period 2015–2023. Three analytical periods were defined based on public health milestones and epidemiological events:

- Pre- vs. post-mandate phase (2015–2016 vs. 2017–2019): To assess the impact of Law 119/2017 introducing mandatory childhood vaccinations.
- Pandemic phase (2017–2019 vs. 2020–2021): To capture the impact of the COVID-19 pandemic on immunization activities.
- Post-pandemic recovery phase (2020–2021 vs. 2022–2023): To assess the recovery in vaccine coverage following the acute phase of the pandemic.

For each vaccine and each region, the average coverage during each subperiod was calculated, and the absolute difference (Δ coverage) was derived. Only regions with available, non-zero data for both subperiods were included in each comparison. Mean differences were computed using available data, even when a year was missing within a subperiod.

### 2.4. Regional Comparisons by Geographic Area

Regions were categorized into three macroareas (North, Center, and South including islands) based on national administrative classifications. Mean Δ coverage values were compared across macroareas using Welch’s *t*-test, which adjusts for unequal variances. Statistical significance was defined as a *p*-value < 0.05. Mean differences and corresponding *p*-values were reported to assess geographic heterogeneity in vaccine uptake trends.

### 2.5. Correlation Between Baseline Coverage and Variation

To investigate whether baseline coverage was associated with subsequent changes in vaccine uptake, we used Pearson’s correlation coefficient (r) to assess the linear relationship between initial coverage values and corresponding Δ coverage for each vaccine and period. Correlations were evaluated separately for each time window. Two-tailed *p*-values were calculated for statistical testing; results were considered significant at *p* < 0.05.

## 3. Results

### 3.1. Temporal Trends in Vaccination Coverage and Joinpoint Analysis

**Figure 1:**
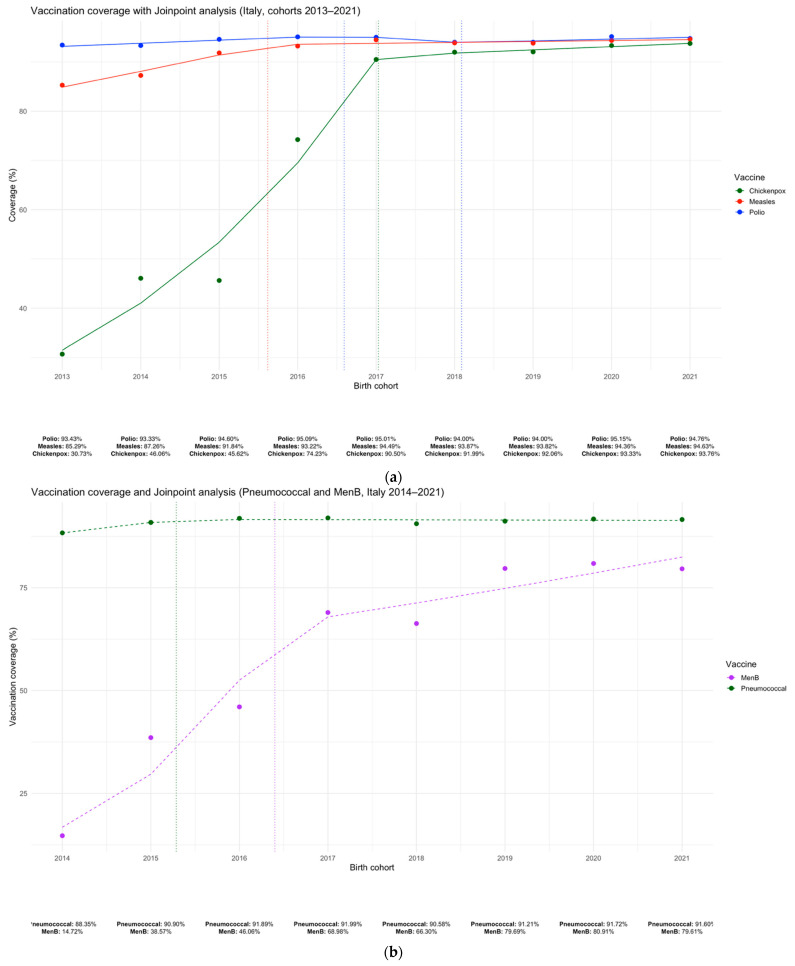
Trends in vaccination coverage with Joinpoint analysis for poliomyelitis, measles, and varicella (**a**), and MenB and pneumococcal vaccines (**b**) in Italy (birth cohorts 2014–2021). Dashed lines indicate joinpoints; slopes represent APCs.

For Polio, coverage remained consistently high, ranging from 93.3% in 2014 to 94.8% in 2021 birth cohort, with no joinpoints identified and an estimated APC of +0.11% (95% CI: −0.10 to +0.32), indicating a stable trend. For Measles, coverage increased from 87.3% in 2014 to 94.6% in 2021, with one joinpoint detected in 2016; a significant rise was observed between 2014 and 2016 (APC = +3.36%, 95% CI: +2.18 to +4.55), followed by a plateau from 2016 to 2021. Chickenpox coverage showed a marked increase from 46.1% in 2014 to 93.8% in 2021. A joinpoint in 2017 marked the shift from a steep growth phase (APC = +28.57%, 95% CI: +17.57 to +40.60) to a stable phase (APC = +0.71%, 95% CI: −7.90 to +10.13) (Figure 1a). For the Pneumococcal vaccine, coverage remained high, between 88.4% and 91.7%, with a non-significant positive trend (APC = +0.31%, 95% CI: −0.04 to +0.66). In contrast, MenB coverage started at 14.7% in 2014 and increased substantially to 79.6% in 2021, with a statistically significant APC of +22.61% (95% CI: +11.02 to +35.41), reflecting rapid uptake of the vaccine following its introduction (Figure 1b). No additional Joinpoints were identified during the COVID-19 pandemic period (2020–2021) for any of the vaccines analyzed.

### 3.2. Regional Trends Across Key Phases (2015–2023)

**Figure 2:**
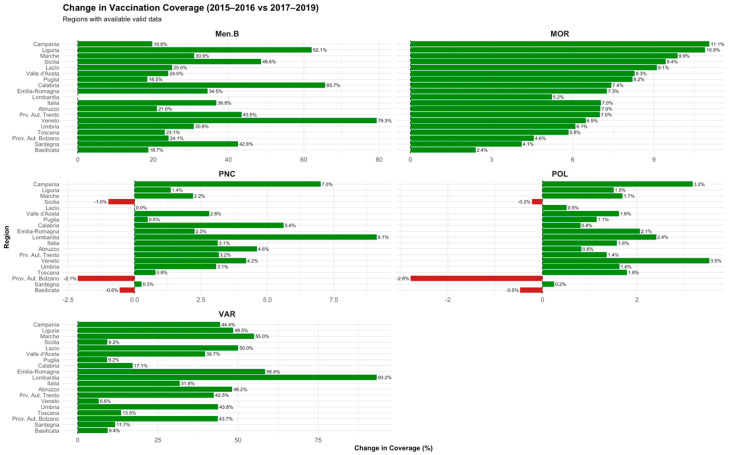
Change in vaccination coverage (2015–2016 vs. 2017–2019). Variation at 24 months by region and vaccine following the implementation of mandatory vaccination (Law 119/2017).

Following the introduction of Law 119/2017, almost all Italian regions experienced substantial improvements in vaccination coverage. Poliomyelitis coverage increased consistently, with gains ranging from +0.24% in Sardinia to +3.53% in Veneto. Measles coverage also showed notable improvements, with regional increases between +2.41% in Basilicata and +11.05% in Campania. Varicella coverage exhibited the most significant growth across regions, with increases from +6.58% in Veneto to an outstanding +93.24% in Lombardia. Similarly, meningococcal B vaccine coverage rose markedly, ranging from +18.48% in Puglia to +79.32% in Veneto. Pneumococcal conjugate vaccine coverage displayed moderate positive changes, with gains ranging from +0.01% in Lazio to +9.11% in Lombardia (Figure 2).

**Figure 3:**
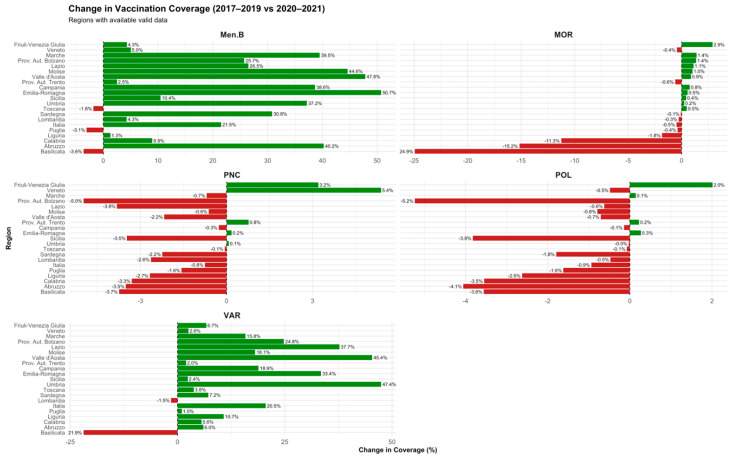
Change in vaccination coverage (2017–2019 vs. 2020–2021). Regional variation by vaccine during the COVID-19 pandemic.

During the COVID-19 pandemic, a decline in vaccination coverage was observed across Italian regions for selected vaccines. Poliomyelitis coverage decreased from −0.03% in Umbria to −5.24% in the Province of Bolzano. Measles vaccination coverage exhibited substantial declines, ranging from −0.08% in Sardegna to −24.94% in Basilicata. Varicella coverage showed heterogeneous patterns, with decreases between −1.51% in Lombardia and −21.92% in Basilicata. Meningococcal B vaccination coverage also declined in several regions, with reductions ranging from −1.82% in Toscana to −3.62% in Basilicata. Pneumococcal conjugate vaccine coverage displayed moderate declines, ranging from −0.07% in Toscana to −4.99% in the Province of Bolzano (Figure 3).

**Figure 4:**
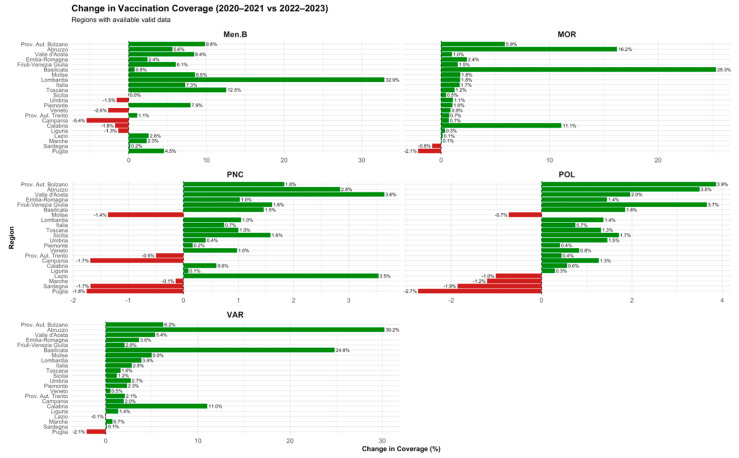
Change in vaccination coverage (2020–2021 vs. 2022–2023). Regional variation by vaccine during the post-pandemic recovery phase.

Following the acute phase of the COVID-19 pandemic, vaccination coverage showed a clear trend towards recovery across most Italian regions (Figure 4). Poliomyelitis coverage increased in the majority of regions, with improvements ranging from +0.29% in Calabria to +3.50% in Abruzzo. Measles vaccination coverage rose substantially, with gains from +0.06% in Calabria to +25.34% in Basilicata. Varicella coverage continued to recover, with increases ranging from +0.13% in Calabria to +30.22% in Abruzzo. Meningococcal B vaccination coverage showed heterogeneous patterns; although most regions recorded positive changes (up to +5.63% in Abruzzo), few regions experienced slight decreases. Pneumococcal conjugate vaccine coverage generally improved, with positive changes reaching up to +2.84% in Abruzzo, despite a few regions showing minor declines.

### 3.3. Geographic Differences in Coverage Trends

Across the three phases analyzed, vaccination coverage trends showed modest geographic variability. During the post-mandate phase (2015–2016 vs. 2017–2019), mean Δ coverage for poliomyelitis was +1.39% in the North, +1.41% in the Center, and +0.79% in the South and Islands (*p* > 0.5 for all comparisons). Measles coverage improved similarly across areas (+7.12% North, +7.73% Center, +7.09% South), while varicella showed a larger gain in the North (+48.35%) compared to the South and Islands (+21.31%), although the difference was not statistically significant (*p* = 0.075). During the pandemic phase (2017–2019 vs. 2020–2021), poliomyelitis coverage declined more sharply in the South and Islands (−2.42%) than in the Center (−0.15%), with a statistically significant difference (*p* = 0.003). Coverage for varicella, measles, MenB, and pneumococcal vaccines showed heterogeneous changes but no significant differences between macroareas. In the post-pandemic recovery phase (2020–2021 vs. 2022–2023), vaccination rates increased across all macroareas, with the most substantial recovery for measles (+8.93% North, +13.96% Center, +6.55% South) and varicella (+13.24% North, +20.15% Center, +6.73% South), though without statistically significant differences (*p* > 0.1). Overall, despite some fluctuations, geographic disparities in vaccine coverage changes remained limited.

### 3.4. Association Between Baseline Coverage and Vaccination Trends

**Figure 5:**
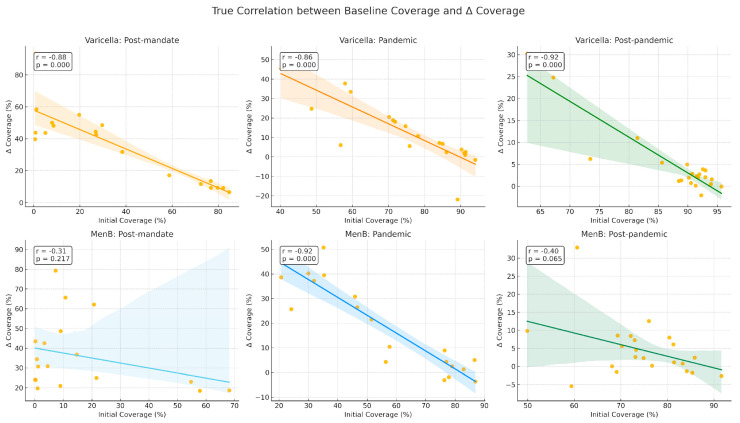
Correlation between baseline coverage and change across periods for varicella and MenB.

Among the vaccines analyzed, varicella consistently exhibited a strong and statistically significant negative correlation across all three periods. Specifically, during the post-mandate phase (2015–2016 vs. 2017–2019), the correlation was r = −0.877 (*p* < 0.001); during the pandemic phase (2017–2019 vs. 2020–2021), it was r = −0.858 (*p* < 0.001); and during the post-pandemic recovery phase (2020–2021 vs. 2022–2023), it reached r = −0.915 (*p* < 0.001), indicating that regions with lower initial coverage experienced greater improvements (Figure 5).

For meningococcal B vaccination, a strong and significant negative correlation was observed during the pandemic phase (r = −0.918, *p* < 0.001), but correlations during the post-mandate and post-pandemic phases were not statistically significant. In contrast, no significant correlations were found for poliomyelitis, measles, or pneumococcal vaccination during any of the analyzed periods, suggesting a more homogeneous pattern of changes irrespective of baseline coverage for these vaccines.

To facilitate interpretation of the findings, Table 1 summarizes the main changes in vaccination coverage across the three defined periods—post-mandate, pandemic, and post-pandemic recovery—for each vaccine included in the analysis. The reported values represent the observed regional ranges of absolute differences in coverage at 24 months of age, highlighting both the direction and magnitude of variation across Italy.

As shown in the table, the most substantial increases in coverage following the introduction of mandatory vaccination policies were observed for varicella and MenB. In contrast, the pandemic period was characterized by marked declines in coverage for measles and varicella in several regions, while the recovery phase showed heterogeneous patterns, with more consistent improvements for measles and varicella, but limited recovery for MenB and pneumococcal vaccines.

## 4. Discussion

This study evaluated changes in vaccination coverage across Italian regions over three key phases: post-mandate (2015–2016 vs. 2017–2019), pandemic (2017–2019 vs. 2020–2021), and post-pandemic recovery (2020–2021 vs. 2022–2023). Following the introduction of mandatory vaccination laws, coverage increased for all vaccines, with varicella showing the greatest rise (+48.4% in the North vs. +21.3% in the South and Islands) and measles improving uniformly (+7.1% to +7.7% across macroareas). During the pandemic, a decline in poliomyelitis (−2.4% in the South) and measles coverage was observed, while MenB coverage continued to improve, particularly in regions with lower baseline rates (r = −0.918, *p* < 0.001). In the post-pandemic phase, coverage levels for all vaccines improved, with varicella (+20.1% in the Center) and measles (+13.9% in the Center) showing the strongest recoveries. Correlation analyses confirmed a strong and consistent inverse relationship between baseline coverage and Δ coverage for varicella across all periods (r = −0.877 to −0.915, *p* < 0.001), while no significant associations were found for poliomyelitis, measles, or pneumococcal vaccines. Overall, these results highlight the effectiveness of legislative actions, the disruptive impact of the COVID-19 pandemic, and good recovery efforts in restoring vaccination uptake. Our results also highlight regional heterogeneity in coverage dynamics, a theme recurrent in Italian public health literature, especially in the context of decentralized healthcare governance.

This study represents one of the first comprehensive evaluations of vaccination coverage trends in Italy extending into the post-pandemic period. By leveraging nationally representative data disaggregated at the regional level, it provides a detailed picture of geographic disparities in vaccine uptake, particularly highlighting persistent differences between northern regions and those in the South and Islands. The findings underscore the critical need for regionally tailored public health strategies to ensure equitable improvements in immunization coverage. Furthermore, this analysis offers important insights into the long-term impact of legislative interventions and the disruptions caused by the COVID-19 pandemic on routine childhood vaccinations, providing valuable evidence to inform future public health planning and policy-making at both national and regional levels.

Consistent with preliminary data suggesting a positive outcome of the mandates [21], our analysis shows an increase in vaccination, especially for measles and the other vaccines combined in the MMR formulation. The increase anticipated the effect of the law, probably due to other contributing factors such as increased disease perception caused by the measles outbreak that occurred in 2017 [22]. Finally, anti-varicella vaccine coverage, which is administered in combination with MMR or alone, increased dramatically over the years, due to several factors, such as the inclusion in the vaccination schedule, the increased availability of the tetravalent formulation (MMR-V), and the inclusion among mandatory vaccines. Similar patterns were observed in other European countries where vaccine mandates have been implemented, such as France and Germany, with increased coverage following legislation, though the effect size and timeline varied [23,24,25,26].

Several statistically significant Joinpoints were identified during 2016–2017, corresponding to a substantial increase in vaccination coverage following the introduction of mandatory immunization policies (Law 119/2017). In contrast, no significant Joinpoints were detected during the COVID-19 pandemic period, suggesting that, at the national level, the decline in vaccination uptake associated with the pandemic did not reach statistical significance. These results are consistent with findings from previous investigations, such as the cohort study conducted by Gómez-Acebo et al. [27], which similarly reported no significant reductions in routine childhood vaccination rates during the pandemic. Nonetheless, a more detailed examination stratified by vaccine type and geographic area revealed declines in coverage for several antigens during the pandemic years. This indicates that, although the overall national trend remained statistically stable, important regional and antigen-specific variations occurred.

Most studies conducted in other countries showed a tendency toward a decrease in vaccination coverage during the pandemic phase, ranging from a slight [28,29,30,31] to a marked decrease [32,33,34,35,36,37]. The decrease in vaccination coverage was likely to be due to a negative impact of the pandemic on immunization services [38], with a slow resumption of their efficiency [39,40,41,42,43]. Previous studies in the United States reported comparable transient declines in measles and varicella coverage during the early months of the pandemic, followed by partial recoveries at 24 months [44,45]. Contrasting the disruption of immunization services is important, since it may lead to a decrease in vaccination coverage, which is a main determinant of the resurgence of VPDs [46,47].

Our findings indicate that the post-pandemic period (2022–2023) was marked by measurable recovery in childhood vaccination coverage across most regions. These data suggest that Italy’s vaccination system demonstrated a certain degree of resilience and responsiveness following the disruption caused by the COVID-19 pandemic. However, the recovery was not uniform throughout the national territory, and disparities persisted across geographic and socio-demographic subgroups [48]. Interestingly, our analysis suggests that regions experiencing the most substantial declines during the pandemic also exhibited some of the strongest recoveries during the post-pandemic phase. This pattern could reflect a compensatory effect, possibly associated with intensified local health interventions, catch-up vaccination campaigns, or increased public awareness following the disruption of routine services. However, recovery was not uniform across all regions, underscoring the need for targeted efforts to address residual disparities in vaccination coverage. Recovery remained incomplete in several areas, particularly for vaccines with historically lower uptake, underscoring the need for sustained investment in catch-up strategies and equitable service delivery. The post-COVID-19 era offers a critical opportunity to leverage insights gained during the pandemic to reinforce public health infrastructure, promote international collaboration in response to health emergencies, and design more resilient and adaptive strategies to address future global health challenges [49,50,51].

Recent studies conducted in other countries have highlighted how parental vaccine hesitancy remained a significant barrier to routine immunization uptake in the post-pandemic era, particularly in relation to influenza and COVID-19 vaccines for children [52,53]. Although our analysis did not directly assess vaccine hesitancy, the observed regional differences in coverage may reflect similar behavioral and psychosocial dynamics. These findings support the need to address trust, perception, and communication issues as part of regional immunization strategies.

Before concluding, we should mention a few limitations of our study, concerning the interpretation of the large regional variations, which may be associated with a series of factors, such as the organization of vaccination services and vaccine hesitancy linked to cultural and social aspects. An analysis of such geographical variations and their determinants goes beyond the scope of this study. Moreover, the ecological and administrative nature of the data limits the possibility of adjusting for potential confounders and of establishing causal relationships. Observed associations between vaccination trends and contextual events—such as the introduction of mandates or the COVID-19 pandemic—should be interpreted as correlations rather than definitive causal effects, as no individual-level data or controlled study design was used. Moreover, as mentioned above, it was difficult to disentangle the effect of specific factors, such as the increase in risk perception due to the measles outbreak and the potential contribution of mandates on the increase in vaccine coverage.

The considerable variability observed across regions and vaccines may be attributed to several context-specific factors. The COVID-19 emergency had an uneven impact on regional health systems in Italy. In the most affected areas, especially in the North during the first wave, the overload of inpatient and outpatient services, reallocation of healthcare workers, closure or reduced functioning of vaccination clinics, and restrictions on public gatherings substantially disrupted immunization activities. These observations align with previous findings highlighting the disproportionate burden experienced by Northern regions—particularly Lombardy—during the early pandemic phase, where excess deaths exceeded 23,000 and the health system faced severe operational stress [16,54]. In other regions, where the healthcare system was less overwhelmed or more flexible in reorganizing services, the impact may have been more limited or more rapidly mitigated [55]. These differences may also explain the observed heterogeneity in post-pandemic recovery trajectories.

Furthermore, unmeasured factors such as socioeconomic disparities and varying levels of vaccine hesitancy across regions may have further influenced vaccine uptake, both during and after the pandemic.

In addition to structural and organizational disruptions, health-related morbidity and mortality due to COVID-19 may have indirectly influenced routine childhood vaccination patterns. During the acute phases of the pandemic (2020–2022), many families experienced illness, hospitalization, or loss of caregivers, which may have limited their ability or willingness to access preventive services such as immunization [51,56,57]. Furthermore, the reallocation of healthcare personnel and the death or long-term absence of healthcare workers due to COVID-19 infection could have contributed to service gaps, particularly in regions already facing resource constraints. Importantly, the perceived prioritization of urgent care and pandemic response over routine services may have also led families and healthcare systems to temporarily de-emphasize childhood vaccinations [2].

Such behavioral and social dynamics, including variations in public trust and health literacy, are difficult to quantify in administrative data but are likely to have played a role in shaping regional trends [58]. Furthermore, confidence intervals were not computed for regional estimates, as the data originate from administrative sources and reflect exhaustive population-level coverage rather than sample-based measurements. While this precludes the quantification of statistical uncertainty at the regional level, the descriptive nature of the analysis remains appropriate for the study objectives. Nevertheless, this represents a methodological constraint inherent to the use of aggregate, non-sampled data, and should be considered when interpreting regional heterogeneity in coverage rates.

## 5. Conclusions

This study focuses on the achievements and challenges of childhood vaccination programs in Italy over the last decade. Mandatory vaccination policies were temporally associated with improvements in coverage for several vaccines, particularly varicella, but also for recommended vaccines such as meningococcal B, while the COVID-19 pandemic, to some extent, disrupted immunization activities, resulting in slight declines for poliomyelitis and measles in some regions. Although signs of recovery were observed during the post-pandemic phase, persistent regional disparities, especially disadvantaging the South and Islands, remain a critical public health concern. The strong inverse correlation observed for varicella between baseline coverage and subsequent improvements across all periods emphasizes the need to prioritize regions with historically lower vaccination rates. However, caution is warranted in interpreting these findings. The use of ecological and administrative data limits the ability to draw causal inferences, and observed associations should be interpreted as correlations rather than direct effects. These findings carry important practical implications. National policies must be complemented by regionally tailored actions that address structural, logistical and sociocultural barriers, reinforce health service delivery, and build public trust in vaccination programs. Investments in surveillance systems and preparedness plans that safeguard routine immunizations during emergencies are equally essential. Ensuring equitable and sustained vaccine coverage nationwide is not only crucial for preventing vaccine-preventable diseases but also for enhancing the resilience of the health system against future public health threats. Targeted, adaptive, and inclusive interventions will be key to consolidating the gains achieved and closing regional gaps in immunization coverage across Italy.

## Figures and Tables

**Table 1:** Summary of changes in childhood vaccination coverage (at 24 months of age) across three periods—post-mandate (2015–2016 vs. 2017–2019), pandemic (2017–2019 vs. 2020–2021), and post-pandemic (2020–2021 vs. 2022–2023)—by vaccine. Arrows indicate general direction of change: ↑ increase, ↓ decrease.

Vaccine	Post-mandate (2015–2016 vs. 2017–2019)	Pandemic (2017–2019 vs. 2020–2021)	Post-pandemic (2020–2021 vs. 2022–2023)
Poliomyelitis	+0.24% to +3.53% (↑ in all regions)	−0.03% to −5.24% (↓ esp. in South)	+0.29% to +3.50% (↑ in most regions)
Measles	+2.41% to +11.05% (↑ broadly)	−0.08% to −24.94% (↓ variable)	+0.06% to +25.34% (↑, esp. in Center)
Varicella	+6.58% to +93.24% (↑, largest increase)	−1.51% to −21.92% (↓ in most)	+0.13% to +30.22% (↑ in most regions)
MenB	+18.48% to +79.32% (↑)	−1.82% to −3.62% (mostly ↓)	−0.5% to +5.63% (mixed)
Pneumococcal	+0.01% to +9.11% (↑)	−0.07% to −4.99% (↓)	−0.2% to +2.84% (slight recovery)

## Data Availability

All data are presented in the current manuscript (text, tables, figures).
